# Determination of the Geographical Origin of Maltese Honey Using ^1^H NMR Fingerprinting

**DOI:** 10.3390/foods9101455

**Published:** 2020-10-13

**Authors:** Chantelle Spiteri, Frederick Lia, Claude Farrugia

**Affiliations:** Department of Chemistry, University of Malta, 2080 Msida, MSD, Malta; chantelle.spiteri.14@um.edu.mt (C.S.); claude.farrugia@um.edu.mt (C.F.)

**Keywords:** Maltese honey, geographic discrimination, mislabeling, multivariate data analysis, machine learning, food authenticity, ^1^H NMR, principal component analysis (PCA), partial least squared-discriminant analysis (PLS-DA)

## Abstract

The price of honey, as a highly consumed natural product, depends on its botanical source and its production environment, causing honey to be vulnerable to adulteration through mislabeling and inappropriate, fraudulent production. In this study, a fast and simple approach is proposed to tackle this issue through non-target one dimensional zg30 and noesypr1d ^1^H NMR fingerprint analysis, in combination with multivariate data analysis. Results suggest that composition differences in sugars, amino acids, and carboxylic acid were sufficient to discriminate between the tested honey of Maltese origin and that of non-local origin. Indeed, all chemometric models based on noesypr1d analysis of the whole fraction honey showed better prediction in geographical discrimination. The possibility of discrimination was further investigated through analysis of the honey’s phenolic extract composition. The partial least squares models were deemed unsuccessful to discriminate, however, some of the linear discriminant analysis models achieved a prediction accuracy of 100%. Lastly, the best performing models of both the whole fraction and the phenolic extracts were tested on five samples of unknown geographic for market surveillance, which attained a high agreement within the models. Thus, suggesting the use of non-target ^1^H NMR coupled with the multivariate-data analysis and machine learning as a potential alternative to the current time-consuming analytical methods.

## 1. Introduction

According to the European Union (EU) legislation, honey is defined as ‘‘the natural sweet substance produced by *Apis mellifera*. Honey consists essentially of different sugars, predominantly fructose and glucose, as well as other substances such as organic acids, enzymes, and solid particles derived from the honey collection” [1]. The main chemical constituent of honey are carbohydrates, adding up to 95% of the dry weight [2] with fructose (38%) and a smaller portion of glucose (31%) as the major sugar component among the 22 different sugar present in the honey composition [3]. A range of amino acids are present as a minor component, constituting mainly proline (80–90% abundancy) and other free amino acids, including glutamic acid, alanine, and tyrosine [4].

The geographical and botanical origin are factors that dictate the price of this widely consumed natural product [5], consequently, the geographic origin of honey should be the same as the area declared on the label [6,7]. Economic profit had caused honey to become an easy target for adulteration, including; deliberate mislabeling of honey origin, the addition of water and sugar to the honey, and feeding of bees with excessive artificial syrup during the nectar collection period [8]. Thus, honey authentication became a longtime concern, which manifested for the need to determine the geographical origin of honey to tackle fraudulent behavior [9], while avoiding unfair competition that may destabilize the market.

The chemical composition of honey varies based on its geographic origin, hence to date, research has mainly focused on investigating the natural physicochemical parameters to establish the floral honey source and/or the origin. As an example, electrical conductivity, sugar, and moisture content, among other parameters, have been studied to identify differences between seasonal Maltese honey [10]. A frequently employed method for honey authentication verification is melissopalynology [11], however, weak pollen flower producers and variations in both honeybees and pollen grain size may result in under-representation of pollen [12]. On a different note, research has also indicated that honey of different geographical origin can be classified based on the variations in the amino acid ratios [13]. The issue remains that when dealing with amino acids in honey, the Maillard reaction needs to be accounted for, since amino acid alterations occur during storage time [14], causing such a parameter to be not ideal for discrimination. Another approach investigated fingerprinting secondary metabolites, such as phenolic compounds, by UV spectra analysis, which identified that phenolic acids and flavonoids could serve as possible markers for geographic authentication and botanical sources [15]. Other studies have utilized numerous NMR techniques such as ^1^H-^1^H correlated NMR spectroscopy (COSY), rotating frame of reference (ROCSY), and nuclear Overhauser effect (NOESY) for structural determination of the complex polyphenols extracted from a rather heterogeneous mixture [16]. 

^1^H NMR spectroscopy gains its popularity for its simplicity, reproducibility, and non-destructive approach [17]. However, apart from the equipment’s costly initial investment, there is a substantial drawback of generating information-rich data that may appear too complex to provide a sufficiently complete analysis by simple techniques [18]. The emerging development of multivariate data analysis and machine learning could overcome these challenges by simplifying the data generated and allowing for both exploratory associations between honey and its botanical origin or its geographical area of production [19,20,21,22]. In fact, numerous studies have demonstrated that principal component analysis (PCA), partial least squared-discriminant analysis (PLS-DA), and canonical discriminant analysis (CDA), among other techniques, allow for the discrimination of different honey types, determine the maturity of the honey, identification of adulteration, and for the confirmation of the presence of specific components [23,24]. A number of different studies have been carried out on the Maltese honey based on physicochemical parameters; these included HMF content, diastase and proline levels, total phenolic content, and sugar composition [25,26]. More recently, a comprehensive study based on the attenuated total reflection mid-infrared (ATR-FT-MIR) spectroscopy showed that it was possible to discriminate and classify local honey from that of non-local samples highlighting the authenticity of the Maltese honey [27].

This study aims to develop chemometric models, from ^1^H NMR data (both noesypr1d and zg30) and multivariate data analysis, sensitive in identifying honey composition differences based on the sample’s geographical origin. Subsequently, both whole honey fraction and phenolic extracts are investigated with the best working models put forward for market surveillance studies. This allows testing the machine learning potential of the models in discriminating between honey samples of Maltese island origin and of non-local origin. 

## 2. Materials and Methods

### 2.1. Solvents and Equipment

All general chemicals of analytical grade were used without further purification. Acetonitrile-d_3_ (C_2_D_3_N) and deuterium oxide (D_2_O) were obtained from Acros Organics (Ferrand, NJ, USA) and Sigma Aldrich (St. Louis, MO, USA), respectively. Methanol was obtained from Hoenywell Fluka (Morris Plains, NJ, USA) and the hydrochloric acid (HCl) was obtained from Sigma Aldrich. Solid-phase extraction (SPE) was performed with SPE Vacuum Manifold standard, a 12-port model, Millipore Millex-GV Syringe Filter Unit 0.22 µm, PVDF, 33mm from Sigma Aldrich and SPE cartridge (1 g silica bonded C-18 resin, Analytical Columns SolGel-1ms™, Trajan Scientific Europe Ltd., Milton Keynes, UK), NMR spectra were obtained by ICON-NMR (Bruker BioSpin, Rheinstetten, Germany).

### 2.2. Sample Collection

**Figure 1:**
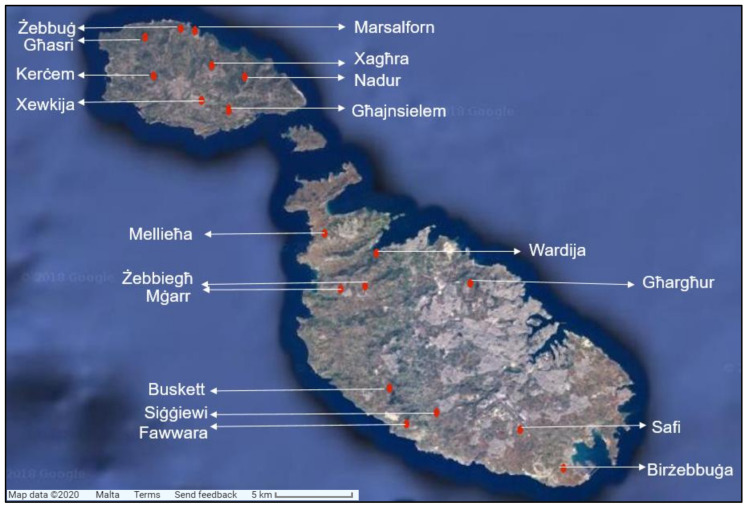
Map of the Maltese Islands indicating the locality of honey samples collection. Source: Google Maps, accessed May 2020.

Proton NMR of zg30 and noesypr1d studies were performed on a total of 84 whole fraction samples (31 samples of Maltese origin, 48 of non-local origin, and 5 of unknown origin). A total of 31 samples were collected from Malta and Gozo (Figure 1), from which 27 samples were collected mostly in 2015, and a small number in 2016, while the remaining four samples (three Maltese and one Gozitan) were collected in 2017. A full list of samples and their origin is presented in supplementary material Tables S1 and S2. Among the 31 samples, seven were bought from the local market, and the rest were collected directly from the beekeepers. The study also investigated the composition of 48 non-local samples used in a recently completed study on the chemical profiling of honey produced in the Maltese islands by Formosa (2017) [27]. A total of 81 phenolic extracts, with the majority extracted from the investigated whole fraction honey samples, were studied with ^1^H zg30 NMR. Five samples of unknown origin were used for market surveillance analysis. All the above-mentioned samples were collected from various localities around the Maltese islands in order to provide a representative sample of the microclimatic conditions and manufacturing and storage techniques that may vary from one sample to another. All the samples were stored in the dark at a temperature of about 20 °C until analyzed.

### 2.3. ^1^H NMR Spectroscopy Acquisition

The analysis was performed on a model AVANCE III 500 MHz NMR spectrometer equipped with a 5 mm ^1^H/D-BB probehead with z-gradient, automated tuning and matching accessory, and a BTO-2000 accessory for temperature control (Bruker BioSpin GmbH, Rheinstetten, Germany). Samples were measured at 300.0 K after a 5 min resting period for temperature equilibration. NMR spectra were acquired using Topspin 3.5 (Bruker). Automated tuning and matching, locking and shimming using the standard Bruker routines, ATMA (automatic tuning and matching in automatic mode), LOCK (frequency-field lock to offset the effect of the natural drift of the NMR’s magnetic field B0) and TopShim, were used to optimize the NMR conditions. Samples were analyzed using the zg30 pulse method and NOESY 1D noesypr1d NMR pulse sequence using a standard resaturation were used for ^1^H NMR. Prior to each run, two dummy scans were obtained, and the final scan resulted in 65,536 data points with the resolution of 0.305 Hz. Acquired with acquisition time and a relaxation delay time of 3.27 s and 4 s, respectively, corresponding to a 90° pulse width of 10 µs. For the whole fraction samples, every sample was run twice with zg30 standard single pulse program for 16 scans and for 100 scans. Water suppression was achieved using one-dimensional nuclear Over-Hauser effect spectroscopy using standard presaturation pulse sequence for 100 scans using the preset 60 dB power level of the presaturation, 1.5 s duration of the presaturation and 300 ms mixing time. Each run had two prior dummy scans resulted in 32,768 data points with the resolution of 0.489 Hz acquired with an acquisition and a relaxation time of ~2.04 s and 4 s, respectively. For ^1^H NMR, the signal-to-noise ratio was calculated using the doublet of doublets peak 2.93–2.89 corresponding to 3-phenyllactic acid, and a signal-to-noise ratio of 3.25:1 and 4.16:1 was obtained for zg30 and noesypr1d pulse sequences, respectively.

#### Whole Fraction and Phenolic Fraction Sample Preparation

The honey samples were heated in a water bath to 25–30 °C while the content was homogeneously mixed. 40 mg of honey was dissolved in 800 µL deuterium oxide (Sigma Aldrich) and transferred to an NMR tube for analysis. In the case of the phenolic fraction preparation, the procedure employed for SPE was similar to that proposed by Michalkiewicz et al. (2008) [28] with minor modifications. For every honey sample, 10 g of honey was dissolved in 100 mL distilled water and stirred for 15 min. A few drops of 6N HCl (Sigma Aldrich) solution was added to the honey solution adjusting the pH to 2.5 at 15.9 °C, followed by filtration through cotton wool to remove any remaining solid particles. The filtrate was passed through an C-18 SPE cartridge previously conditioned with 25 mL methanol (Fluka) followed by 25 mL of pH 3 HCl (Sigma Aldrich). After passing the filtrate, the cartilage was placed under vacuum and washed with 50 mL of pH 3 acidic water (HCl) followed by washing with 50 mL of distilled water to remove polar constituents of honey, such as sugars. Lastly, the column was eluted with 20 mL methanol to recover substances adsorbed to the column. The eluted solution was filtered through a Millipore Millex-GV Syringe Driven Filter Unit hydrophilic polyvinylidene fluoride (PVDF) membrane with 0.22-µm pores for the removal of sugar particles. The resulting methanol fraction was dried under a gentle nitrogen stream, reconstituted into 1 mL of methanol and stored at a temperature of 4 °C until further analysis. Once needed for analysis, the extracts were left at room temperature and a volume of 200 µL from each extract sample was transferred to another vial and dried under nitrogen at a temperature not exceeding 40 °C. Then, 400 µL of acetonitrile-d_3_ (Acros Organics) was added to the extracted sample and vortexed for about 30 s followed by transferring of the contents to an NMR tube. Another 400 µL of acetonitrile-d_3_ was added to the same vial, and the process was repeated in order to obtain any remaining extract, which was then added to the same NMR tube for analysis. 

### 2.4. Spectral Preprocessing and Pretreatment

The NMR spectra were acquired through TopSpin™ version 5. For all the spectra, phasing and baseline undulations were manually corrected over the integrated regions, and the chemical shift position was calibrated through an internal reference by setting the tetramethylsilane (TMS) peak to 0. In addition, the solvent peaks of D_2_O (4.80 ppm) and acetonitrile-d_3_ (1.94 ppm) were also used as an external reference for the whole fraction and phenolic extract NMR spectra respectively. The region of interest, from 0 to 10 ppm, was transferred into a separate spreadsheet for both the whole fraction and the phenolic extract spectra. Such spreadsheets were exported as an ASCII file (.csv) and imported into The Unscrambler^®^ X 10.3 (CAMO Software Oslo, Norway) for subsequent mathematical data processing. The preliminary data was transformed, and the number of data points (variables) was reduced by a reduction factor of 10 without losing resolution. The noise was further reduced by the moving average smoothing technique, set with a segment size value of 5, followed by baseline correction of the spectra. Subsequently, on the pretreated spectral data; the normalization, detrending, deresolve, multiplicative scatter correction (MSC), standard normal variance (SNV), 1st and 2nd Gap derivatives with a segment size of 5, 1st and 2nd Savitzky-Golay transformations with a gap size of seven points and a polynomial order of two functions were applied.

### 2.5. Data Analysis

Once the transformations were complete, the artificial peak of the deuterated solvent was removed to avoid its influence in generating misleading results in the following analysis. Unsupervised PCA was carried out on every spectral transformation for pattern recognition and outlier identification. In regression analysis, the supervised PLS technique was performed. The excluded row validation method was used to split the sample into two groups, one set to train the model and the second set to measure the resulting model’s error. The two sets had equal sample distribution from both geographical origins through the excluded row validation method, hence, representative of the entire sample set. This approach was anticipated to increase the model’s robustness and predictability reliability. Since the number of latent variables was quite large, Variable Influence on Projection (VIPs) parameters were used to select a subset of variables of real importance. Those variables with a VIP less than 1.0 were removed, while the remaining variables were deemed significant for revaluation by PCA and PLS-DA, using The Unscrambler^®^ X 10.3 software.

To ensure that the conclusions drawn from the model were justified, 69 and 66 samples from the whole fraction and phenolic dataset respectively were chosen through leave-one-out-method internal validation. Since internal validation tends to give an overoptimistic result, external validation was also essential for double cross-validation. The sample sizes were determined by Venetian blinds cross-validation, where the samples were divided into non-local and local samples, and then every 5th and every 3rd sample was selected, respectively, to have the prediction samples representing the whole data set. The remaining samples were used for training and testing data sets. The goodness-of-fit of PLS was assessed from the percentage of variability explained in X and in Y, and the sensitivity of the model on a specific number of latent variables was assessed by Predictive Residual Sum of Squares (PRESS). PLS was done, followed by discriminate analysis on the scores obtained from the processed data matrix. A dummy binary-coded one-dimensional response vector was created to tackle classification problems and an assumption was made in which the values smaller than 0.5 were rounded as 0 and values greater than 0.5 were rounded as 1. In such a case, a value of 0 represents local honey while a value of 1 represents non-local honey.

#### Stepwise Linear Canonical Discriminate Analysis (SLC-DA)

**Figure 2:**
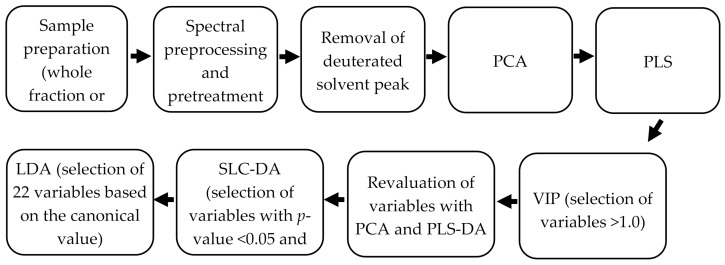
Summary of the stepwise data analysis process. The samples are first prepared for NMR analysis, the generated spectra were preprocessed and pretreated, and the deuterated solvent peaks were removed from the resulting spectral transformation. Sequentially, PCA and PLS were performed, followed by a reduction of variables through VIP, and the resulting spectra was revaluated with PCA and PLS-DA. Lastly, the variables were further reduced through SLC-DA and the selected 22 variables (11 variables with the highest and 11 variables with the lowest canonical value) were analyzed by LDA.

A SLC-DA method was done by JMP^®^, Version 10 SAS Institute Inc., Cary, NC, USA, 1989–2020) to decrease the likelihood of overfitting. The selected variables had a p-value smaller than 0.05 and the largest *F*-ratio, representing the highest correlation with the response. This process reduced the number of variables to 77. Furthermore, the 77 variables were reduced to just 22 variables (eleven variables with the highest canonical value and another eleven variables with the lowest canonical value) based on their standardized coefficient scores. The resulting spectrum was used for LDA analysis with The Unscrambler^®^ X 10.3. Being a supervised technique, the samples were arranged in ascending order and 20% of the samples were selected by the Venetian blind method, creating two sample sets for prediction analysis. For the phenolic extracts, 61 samples were used as the testing set, and the remaining 20 samples (9 local, 10 non-local, 1 market surveillance) were used for prediction analysis, while for the whole fraction samples, 64 samples were used as the testing set, and the remaining 20 samples (7 local, 8 non-local, 5 market surveillance) were used for prediction analysis. Double cross-validation could then be performed on sample sets representative of the two populations. A summary of the stepwise data analysis process described above is depicted in Figure 2. 

## 3. Results

**Figure 3:**
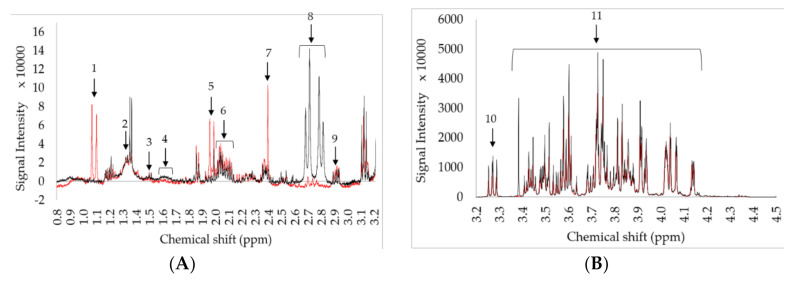

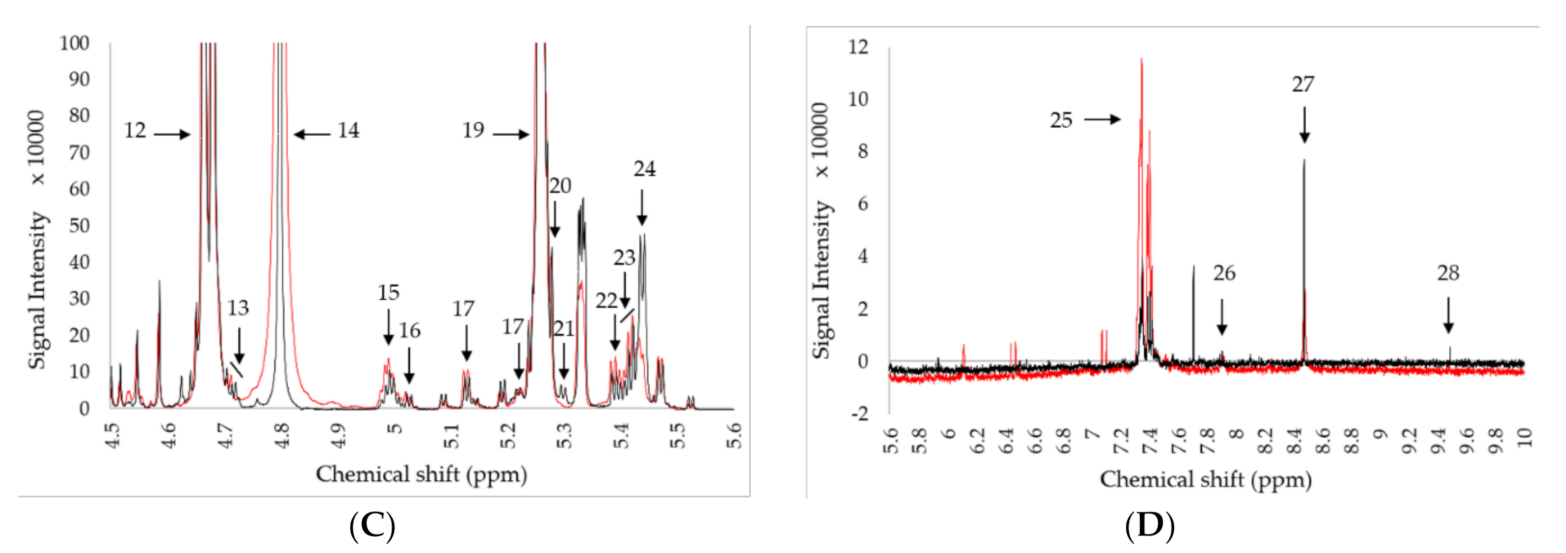
A zoom on the ^1^H NMR whole fraction spectra observed between; (**A**) 3.2–0.8 ppm, (**B**) 4.5–3.2 ppm, (**C**) 5.6–4.5 ppm, and (**D**) 10–5.6 ppm. The red and black spectral lines correspond to non-local and local samples, respectively. The arrows indicate the identified compounds listed in Table 1.

The spectrum consists of many overlapping signals, in which one can work around it through signal processing and multivariate methods. A general whole fraction spectrum and the main chemical signals identified are displayed in Figure 3 and Table 1 respectively while an overlay of the general phenolic extract spectrum for both local and non-local origin is provided in the supplementary (Figure S1). Unlike the whole fraction spectrum, the phenolic extract spectrum were solely used for fingerprint analysis, and thus, the peaks within the extract spectrum were not identified individually. More so, an overlay of the zg30 and noesypr1d pretreated spectra is provided in the supplementary (Figure S2), highlighting the difference in the noise to signal ratio between the two types of pulse programs.

### 3.1. PCA Visualisation of Pretreated Data

**Figure 4:**
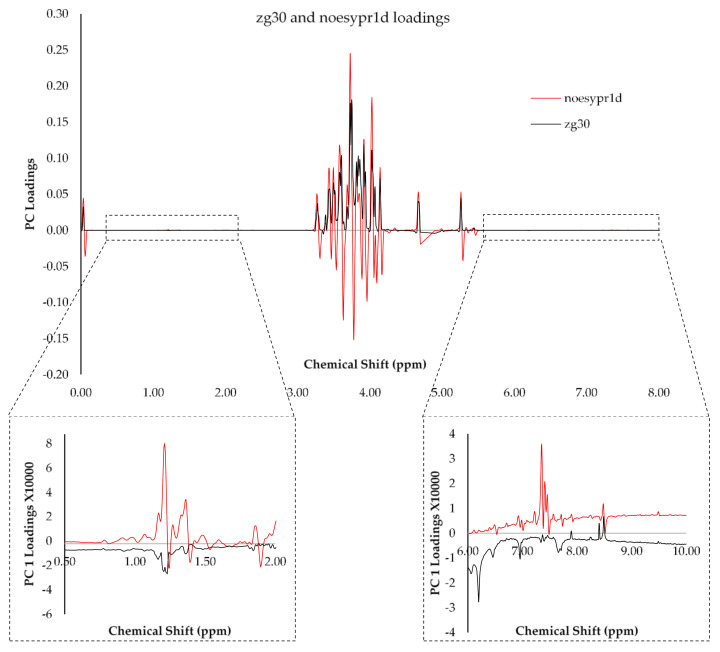
First principal component loading plots observed for zg30 (black) and noesypr1d (red) ^1^H NMR spectra after deresolve for the complete honey fraction.

Prior to developing the chemometric models, eleven different kinds of spectral pretreatments were applied for every spectral data set. The pretreatment reduces the noise while enhancing smoothing, account for scattering and correct baseline and linear trends. Awareness of the intervariable relationships is essential when conducting these reduction methods as treating the variables in isolation may result in loss of important information [31]. PCA of all the spectral pretreatments indicated that none of the samples was considered as a gross outlier and only weak clustering of the samples was obtained. The percentage explained variance by the first two principal components, for both zg30 and noesypr1d, always accounted for 76–94% of the total variance, an indication that these two components contribute greatly to the total variance and that there is little numerical noise from the data. As expected, the percentage variability of the raw data PCA for zg30 and noesypr1d pulse program was lower than for the spectral pretreatment methods. Furthermore, analysis of the loading plots for PCA obtained using the two pulse programs revealed that the noesypr1d had larger loadings between the 4.00–3.00 ppm suggesting an improvement with respect to the variability explained with respect to the different sugars present. Furthermore, the loadings observed corresponding to minor constituents in the honey, such as amino acids observed at between 1.50–1.00 ppm and 3-phenyllactic acid, phenylalanine observed at 7.41–7.34 ppm were much higher for the noesypr1d (Figure 4). Unfortunately, the PCA transformations as obtained for the phenolic extracts had low principal components (56% to 69%) suggesting that most of the variables were noise. 

### 3.2. Determination of Geographical Origin by PLS-DA

The 16 and the 100 scan model on the whole honey fraction had little discrepancy in their results, with the latter model performing slightly better on most occasions. Only two of the 100 scan model transformations managed to correctly discriminate 70% samples. On the contrary, almost all the noesypr1d models achieved 70 or 80% accuracy and were able to correctly classify the samples to their respective geographical origin. The PLS-DA models on phenolic extracts performed poorly since these were only able to correctly classify the samples less than half of the time. Moreover, there was a large discrepancy between the training percentage accuracy and the prediction percentage accuracy, indicating that this model was overfitting.

### 3.3. Discrimination by Variable Influence on Projection (VIPs)

The disadvantage of having a profile study is that not all of the collected data is significant and since the amount of X-variables is much larger than the amount of samples, data reduction techniques needed to be employed. Performing PLS analyses on the variables with a VIP value greater than 1.0 demonstrated an improvement in the model’s discrimination ability for some of the transformations. 

#### VIPs on the Whole Fraction and Phenolic Extracts

The outcome of the treated NMR spectra as obtained from the 16 and 100 scans were very similar, therefore, from here onwards, only the 100 scans were analyzed further. In continuation with the previous PLS results obtained, noesypr1d models exhibited better performance than the zg30, with the majority of the transformations achieving a prediction accuracy of 80%. The following zg30 100 scans transformations; MSC, SNV, and Normalize had the highest percentage accuracy but with a relatively low percentage variation in Y (the geographical origin). In addition, the percentage accuracy of zg30 and noesypr1d between the two cross-validation methods did not vary greatly, hence suggesting that the regressions models were not overfitted. A confirmation that the variables removed were indeed redundant was provided by PCA of the selected variables as the percentage of variance explained was generally improved slightly for both the zg30 and noesypr1d. 

The typical PLS loading plots of both zg30 and noesypr1d indicate that the variables with the most intense region, within the range of 4.14–3.24 ppm, 4.66–4.63 ppm, and 5.26–5.23 ppm, were overlapping with monosaccharides and disaccharides. Although the sugar composition in honey is probably the most influential component to differentiate between the samples, such spectral regions are similar throughout all the samples, hence ideally, these are not used to distinguish between the geographical origin. The high-intensity region at the 4.14–3.22 ppm range consisted also of proton signals from other compounds as the CH_3_ multiplet of alanine at 3.85–3.75 ppm and the ethanol multiple CH_2_ at 3.66–3.56 ppm [29]. The first and second PC were also influenced to a minor extent by several individual peaks, such as 4.98 ppm representing isomaltotriose, kojibiose, and/or isomaltose protons, 5.31 ppm representing the chemical shift of kojibiose, maltose, or the anomeric proton of the glucose residue of sucrose [30], and 5.41 ppm obtained from sucrose (H_1_) [32]_._ MSC and SNV of zg30 100 scans transformations, together with MSC of noesypr1d, exhibited two other peaks at 5.11 ppm and 5.23 ppm representing kojibiose and maltulose, or nigerose protons [33] respectively.

**Figure 5:**
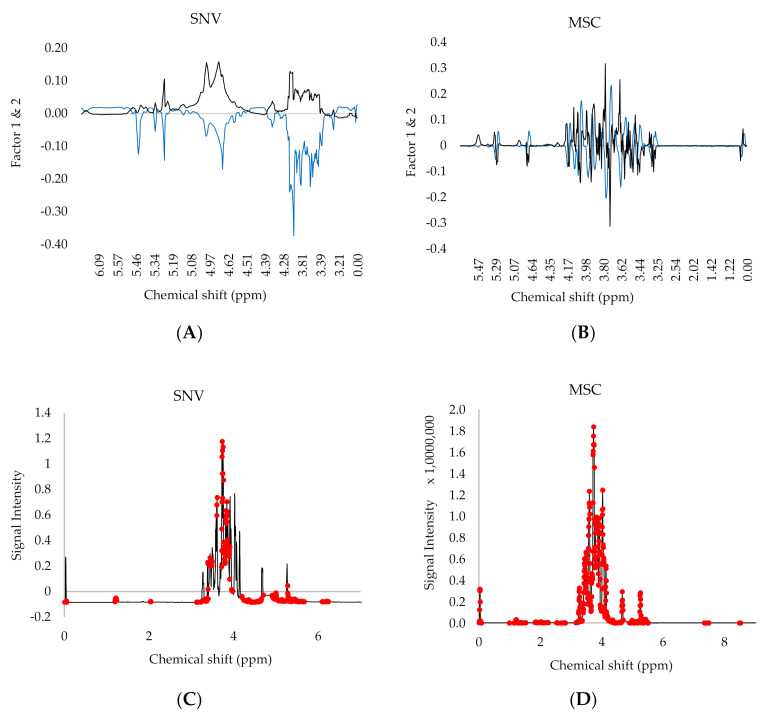
PLS VIP loading plots. The PLS VIP loading plots of (**A**) zg30 100 scans (SNV) and (**B**) noesypr1d (MSC) of the whole fraction and (**C**,**D**) their corresponding variables having a VIP >1 marked in red.

Finally, it was observed the SNV transformation of the zg30 also exhibited minor peaks between the 6.15–7.63 ppm region as important variables influencing the scattering, the presence of which suggests the presence of aromatic protons such as those of polyphenols, 5-HMF, phenylacetic acid, tyrosine, 3-phenyllactic acid and phenylalanine [30]. A closer inspection of the VIP loading plot, as shown in Figure 5(A,B), reveals a number of peaks within the 3.15–0.2 ppm and the 8.5–5.9 region visible. The minor components within such regions include; isoleucine CH_3_ at 0.93 and 1.1 ppm, leucine CH_3_ d at 0.95 ppm and 0.97 ppm, valine CH_3_ d at 0.99 ppm and 1.04 ppm, ethanol CH_3_ at 1.18 ppm, lactic acid C3H_3_ d at 1.35 ppm, alanine CH_3_ at 1.46 ppm, proline at 2.05 ppm, 2.17 ppm and 2.43 ppm [33], acetic acid C_2_H_3_ s at 2.07 ppm, methylglyoxal CH_3_ at 2.25 ppm, succinic acid C_2_H_2_ and C_3_H_2_ at 2.68 ppm, citric acid dd at 2.79 ppm and 2.94 ppm [34], 5-HMF at 6.68 ppm, 6.90 ppm and 7.54 ppm, phenylalanine aromatic region at 7.10 ppm, 7.31 ppm and 7.39–7.35 ppm, formic acid HC at 8.31 ppm and HCO_2_H singlet at 8.45 ppm [30] and further aryl signals at 7.41 and 7.66 ppm [35]. 

Regarding the phenolic extract, further variable reduction did not improve the predictive ability of the model. In fact, the testing percentage accuracy remained about the same as that obtained by the previous PLS-DA.

### 3.4. Goodness of Fit

Goodness of fit models were taken into consideration to prevent the bias associated with statistical analysis interpretation. In all instances, the p-value of van der Voet T^2^ was greater than 1, rejecting the null hypothesis, suggesting that there was a significant difference between the proposed model and the model obtained. Furthermore, the goodness of fit of PLS-DA were investigated by PRESS, the percentage variability as explained in X, percentage variability as explained in Y, and also in terms of the number of factors extracted. For the model to be regarded suitable, the PRESS value and the number of extracted factors should be low while the percentage of variability, as explained in Y, should have a high value. Table 2 shows that the transformations obtained from noesypr1d had a better performing model owing to the smaller difference in the percentage of variability explained in terms of X and Y than for zg30. 

### 3.5. Determination of Geographical Origin by SLC-DA and LDA

**Figure 6:**
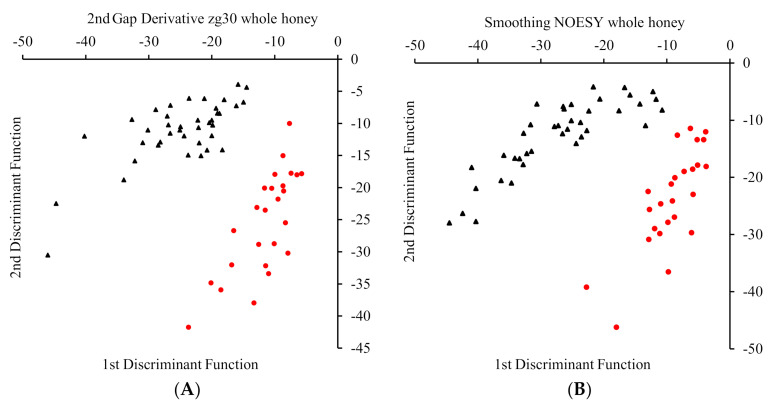
The clustering of the samples into two distinct groups. The separation of the samples as described by the first and second discriminant functions during (**A**) zg30 100 scans for 2nd Savitzky Golay Derivative and (**B**) noesypr1d for smoothing of the whole fraction. The black triangles indicate non-local origin, whereas the red dots indicate local origin.

The model is more likely to overfit when the ratio of the number of variable to the number of samples is small. While SLC-DA reduced the variable to 77 to enhance the model’s performance, such a huge reduction may be regarded as a loss of valuable information, yet, the spectral profile of the honey samples was, in general, retained. Moreover, to perform LDA, the 77 variables were reduced to 22 variables. To justify the discriminatory importance of the 22 variables, these were plotted on the whole spectrum and the majority of which were present either at peak signals or in between, suggesting the retention of the data sensitivity. The testing accuracy of zg30 and noesypr1d LDA models ranged from 81–100% while all the noesypr1d models with the exception of one, had an accuracy of 80% or higher as shown in Table 3. The clustering of the samples into two classes is depicted in Figure 6.

#### LDA on Phenolic Extracts

**Figure 7:**
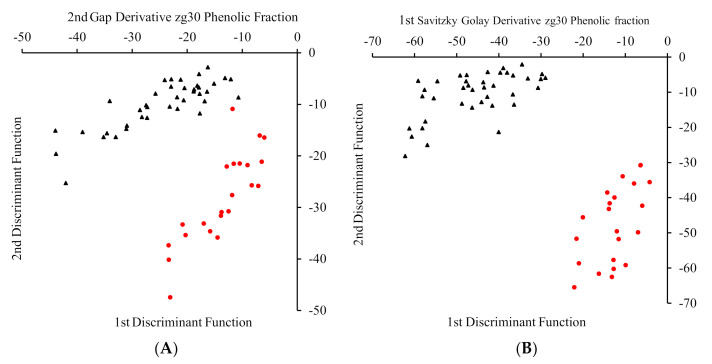
The clustering of the samples into two distinct groups (**A**,**B**). The separation of the samples as described by the first and second discriminant functions during zg30 120 scans LDA transformations of the phenolic extract. The black triangles indicate non-local origin, whereas the red dots indicate local origin.

The modeling power was greatly enhanced for the phenolic extracts upon LDA treatment with a testing accuracy of 81–100% and a prediction accuracy of 63–100% (Table 4). In fact, Figure 7 represents two of the best performing LDA models, whereby the local and the non-local samples are neatly arranged into two distinct clusters. Additionally, as indicated in Table 4, zg30 120 scans and 1200 scans showed similar results, suggesting that pretreatment of the spectra is sufficient to reduce noise data that would otherwise be removed through longer NMR acquisition time. 

Based on our hypothesis, phenolic extracts composition provided a simpler NMR spectrum than the zg30 100 scans and 1D NOESY counterparts, hence simplifying the machine learning process and allowing for better discrimination between the two origins of the honey. The main common variables selected by LDA treatment were within the ranges 1.15 ppm, 1.40–1.37 ppm, 1.62 ppm, 2.89–2.49 ppm, all of which are due to –OH from alcohol or from -CH of alkanes. Other important variables were extracted at 3.33 ppm, 3.95–3.77 ppm, which represent the –OCH_3_ group of the phenolic structures such as 3-methylquercetin, ferulic acid, pinoresinol, syringic acid, vanillic acid, and vanillin. The chemical shifts within the range of 5.70–5.44 ppm are a representation of alkene protons; hence phenolic compounds such as abscisic acid, caffeic acid, chlorogenic acid, cinnamic acid, and ferulic acid are important for discrimination between geographical origin. The aromatic region is attributed to numerous variables selected between the range of 8–6 ppm. Finally, a small number of variables with discriminatory ability were selected within the 9.65–9.55 ppm as this chemical shift is due to the presence of the aromatic –OH group [35]. The above highlights that despite the drastic reduction in the number of variables, the remaining variables considered important by LDA treatment are those representing alcohols/phenols (as was expected from spectra of phenolic extracts). Therefore, emphasizing that the discrimination put forward through LDA treatment is based on differences in the chemical composition of the honey samples rather than due to noise.

### 3.6. Investigating Model Consistency through Market Surveillance

The best performing models were tested with five samples of unknown geographical origin to determine the model’s relevance of use for market surveillance. The models were in general agreement with the overall classification of the samples. The samples labeled A2 were listed by the models as local, samples A3 and A5 were classified as non-local, while sample A4 was listed as non-local except for the LDA noesypr1d model, which classified it as local. A possible reason for the distinctive results obtained by such a model might be because the data gathered by the noesypr1d NMR technique is already focusing on a smaller set of variables as compared to ^1^H zg30. Therefore, the variable reduction techniques could have resulted in the extensive removal of variables, some of which important for the discriminatory classification of the samples. The models’ reliability on sample A4 was checked from the best performing models of the phenolic extract (LDA zg30). The result of which classified it as non-local, agreeing with the models based on the whole fraction. The percentage agreement within the transformations of a model for each sample is tabulated in Table 5. From the models obtained, we were unable to determine the origin of A1 samples suggesting that this sample was adulterated and we were unable to determine the exact origin of such sample. Models that account for sample adulterations need to be produced, however, this was outside the scope of the study.

## 4. Discussion

This preliminary study’s potential is to provide an indication of the success in detecting fraudulent products, mainly falsification of the honey’s origin through the application of chemometric models. In unsupervised PCA models, there was no significant clustering, hence, classifying these models were unsatisfactory in discriminating the geographical origin. In comparison, the supervised techniques were more reliable for both zg30 and noesypr1d proton NMR while also noticing that different pretreatment methods influence the sensitivity of the model differently. Although PLS-DA on both the pretreated and the VIP spectra for both zg30 and noesypr1d models obtained similar percentage accuracy in classification, overall noesypr1d models performed better. This serves as a clear indication that the NMR pulse methods play a role in influencing the signal to noise ratio. In fact, the classification of the raw noesypr1d data in comparison to the zg30 raw dataset performed better, achieving a 100% prediction. It is thus suggested that such a high prediction accuracy is attributed to the higher signal to noise ratio achieved through the noesypr1d pulse method and by detecting peaks that were completely alienated in the zg30 pulse method. Lastly, LDA models provided further enticing results as the remaining variables were able to correctly classify the samples to a higher percentage accuracy. 

A relatively small number of variables were deemed to be important in discriminating between Maltese honey from non-local honey samples. Among these variables were the major intensive signals dominating withing the 4.18–3.24 ppm region, which is contributed by the monosaccharides and disaccharides. The remaining chemical shifts with high discriminatory power were mainly within the 3.2–0.5 ppm and 8.5–6.0 ppm range. The former reveals the presence of several amino acids, including alanine, valine, leucine, and proline, which are derived from the salivary secretions during the conversion of nectar into honey. The latter range is due to the aromatic region of compounds such as phenylalanine, phenylacetic acid, polyphenols, and 5-hydroxymethylfurfural.

The complexity of the whole honey fraction may render the identification of the compounds difficult, therefore, phenolic extracts were considered to increase the sensitivity towards discrimination. In fact, phenolic acid and polyphenols have been suggested as possible discriminatory markers for the identification of both the botanical and geographical origins of honey [36,37]. Unfortunately, with regard to the phenolic extracts, both the unsupervised PCA and PLS-DA models were unsuccessful in discrimination. A possible explanation is that the samples studied in the referred literature had samples from different continents, whereas all the samples for our study were obtained from Europe, rendering them insufficiently different for discrimination analysis. Additionally, although the poplar species in Europe vary in pollen composition, these have similar flavonoid patterns in honey, which may also contribute to the lack of discrimination [38]. On a more positive note, the LDA model was successful in classifying most of the sample tests correctly. Some of the best performing models developed were taken further for market surveillance analyses, in particular LDA models of zg30 and all noesypr1d models for the whole fraction. All of which reached an agreement regarding the classification of the five samples provided with unknown geographical origin. 

Throughout the entire data interpretation, it was essential not to take any value in isolation, especially when considering the relatively small sample size used in this preliminary study. To reduce errors, the samples were tested with the unsupervised technique to remove any outlier should this be detected. More importantly, to avoid the problem where a model could overfit even though the percentage accuracy was high, the difference between the training and the prediction percentage accuracy was considered. The small differences between the two indicated that the model was indeed not overfitting. It can thus be concluded that careful development and diligent interpretation of the multivariable statistical models serve as a fast, and reliable tool to make geographical predictions with relatively little effort as compared to manual procedures. 

## 5. Conclusions

Extracting information on the composition of honey samples through non-targeted ^1^H NMR analysis in combination with chemometrics generates a powerful tool capable of discriminating between samples of the Maltese islands and those of non-local origin. Overall, noesypr1d experimental technique performed better than zg30, while analysis of the data obtained through phenolic extracts was unable to discriminate except for the LDA models whereby a 100% prediction accuracy was obtained in some of the models. In addition, such models identified a smaller set of variables, mainly involving sugars, amino acids, and carboxylic acids to be responsible for discriminating between the two honey types. It can be concluded that the general agreement within the models during market surveillance indicates that the technique in this study can serve as a suitable alternative for discriminating between honey of two origins in questions.

## Figures and Tables

**Table 1:** Identified peaks obtained by ^1^H noesypr1d NMR within the 9.5–0.5 ppm region [5,17,29,30].

Multiplicities	Chemical Shifts (ppm)	Peak Identity
d	1.10–1.07	Valine
d	1.32	H_3_-lactic acid [27]
d	1.50	CH_3_-alanine
m	1.68–1.56	Leucine
s	2.07	Acetic acid
m	2.28–2.01	Proline
s	2.40	Succinic acid
dd	2.82–2.68	Citric acid
dd	2.93–2.89	3-phenyllactic acid
	3.29–3.24	H_2_-β-glucose
	4.17–3.24	Di-/mono-saccharides
d	4.70–4.64	H_1_-β-glucose (anomeric)
d	4.72–4.70	Isomaltose, Isomaltotriose, Gentiobiose, Maltose, Panose
s	4.80	D_2_O
	4.99–4.98	Isomaltose, Isomaltotriose, Panose, Kojibose
d	5.02–5.01	Raffinose [5]
d	5.13	Kojibose
d	5.24	Maltulose
	5.25–5.27	H1-α-glucose, Gentobiose
d	5.28	Maltulose, Melibiose
d	5.30	Turanose
d	5.41–5.39	Maltose, Sucrose, Panose, Kojibose
d	5.42	Kestose, Raffinose
d	5.44	Melezitose, Kojibiose
	7.41–7.34	3-phenyllactic acid, Phenylalanine
	7.89	Benzoic acid
s	8.45	Formic acid
s	9.49	HMF

The range between 4.17–3.24 ppm is not identified individually due to the large sugar units overlap, causing difficulty in discrimination. Abbreviations: s, singlet, m, multiplet, d, doublet, dd, double of doublets.

**Table 2:** A summary of the percentage accuracy of the PLS models obtained for zg30 and noesypr1d spectra transformation on the whole fraction using variables with a VIP value >1.0.

Pretreatment	VIP >1.0 Data Set for zg30 NMR			VIP >1.0 Data Set for Noesypr1d NMR		
	Train	Test	Predict	Train	Test	Predict
Smoothing	63.77	60.87	50	75.36	71.01	80
Baseline	62.32	62.32	50	76.81	71.01	80
Deresolve	62.32	60.87	50	82.61	75.36	80
Detrend	62.32	50.72	60	75.36	72.46	80
MSC	68.12	69.57	70	76.81	71.01	70
SNV	71.01	69.57	70	76.81	72.46	80
Normalize	71.01	56.52	70	78.26	72.46	80
1st Gap	63.77	60.87	60	78.26	73.91	80
2nd Gap	62.32	59.42	60	75.36	75.36	80
1st SG	91.30	78.26	60	79.71	69.57	60
2nd SG	63.77	59.42	60	78.26	75.36	70
Raw Data	65.22	53.62	60	75.36	72.46	100

**Table 3:** The percentage accuracy summarising the performance for the different spectral pretreatments for zg30 100 scans and noesypr1d LDA models on the whole honey fraction.

Pretreatment	zg30 % Accuracy		Noesypr1d % Accuracy	
	Test	Predict	Test	Predict
Smoothing	90.63	80	100	100
Baseline	93.75	80	100	80
Deresolve	90.63	93.33	100	93.33
Detrend	85.94	93.33	90.63	40
MSC	81.25	66.67	96.88	80
SNV	82.81	66.67	90.63	86.67
Normalize	95.31	93.33	96.88	80
1st Gap Der.	93.75	73.33	95.31	86.67
2nd Gap Der.	96.88	100	100	93.33
1st SG Der.	89.06	93.33	100	93.33
2nd SG Der.	100	100	96.88	86.67
Raw Data	89.06	60	98.44	80

**Table 4:** The percentage accuracy summarising the LDA performance for the different spectral pretreatments for zg30 models on the phenolic extracts with the internal and external cross-validation methods.

Pretreatment	120 Scan % Accuracy		1200 Scan % Accuracy	
	Test	Predict	Test	Predict
Smoothing	98.36	84.21	93.44	84.21
Baseline	98.36	84.21	95.08	84.21
Deresolve	98.36	89.47	91.80	89.47
Detrend	95.08	73.68	95.08	78.95
MSC	81.97	84.21	86.89	73.68
SNV	83.61	78.95	95.08	89.47
Normalize	93.44	84.21	96.72	63.16
1st Gap Der.	100	89.47	100	100
2nd Gap Der.	98.36	94.74	100	89.47
1st SG Der.	100	94.74	95.08	94.74
2nd SG Der.	100	89.47	98.36	89.47
Raw Data	98.36	94.74	100	78.9

**Table 5:** The predicted classification of five samples with unknown geographical origin. The whole fraction by zg30 and noesypr1d were only analyzed by the best working models. Value 0 and 1 represent local and non-local origin, respectively.

Model	Sample Code	Whole Fractions Predicted origin											% of Transformations Predicting the Respective Geographical Origin	
		Smoothing	Baseline	Deresolve	Detrend	MSC	SNV	Normalize	1st Gap Der.	2nd Gap Der.	1st S. Golay Der.	2nd S.Golay Der.	0	1
LDA zg30 100 scan	A1	0	1	1	0	0	1	0	0	1	1	0	54.5	45.5
	A2	0	0	0	0	0	0	0	0	0	0	0	100.0	0.0
	A3	0	1	1	1	0	1	1	1	1	1	1	18.2	81.8
	A4	1	1	1	1	0	1	1	1	1	1	0	18.2	81.8
	A5	1	1	1	1	1	1	1	1	1	1	1	0.0	100.0
PLS-DA NOESY	A1	0	0	0	0	0	0	0	1	1	1	1	63.6	45.5
	A2	0	0	0	0	0	0	0	0	0	0	0	100.0	0.0
	A3	1	1	1	1	1	1	1	1	1	1	1	0.0	100.0
	A4	1	1	1	1	1	1	1	1	1	1	1	0.0	100.0
	A5	1	1	1	1	1	1	1	0	0	0	0	36.4	63.6
PLS-DA noesypr1d VIPs	A1	0	0	1	0	0	0	0	1	1	1	1	54.5	45.5
	A2	0	0	0	0	0	0	0	0	0	0	0	100.0	0.0
	A3	1	1	1	1	1	1	1	1	1	1	1	0.0	100.0
	A4	1	1	1	1	1	1	1	1	1	1	1	0.0	100.0
	A5	1	1	1	1	1	1	1	0	0	0	0	36.4	63.6

## Supplementary Materials

The following are available online at https://www.mdpi.com/2304-8158/9/10/1455/s1, Table S1: A list the location and the botanical origin of each local honey sample used in this study; Table S2: A list the location and the botanical origin of each non-local honey sample used in this study; Figure S1: A zoom on the 1H NMR phenolic extract spectra observed between; (A) 3.0–0.0 ppm, (B) 5.0–3.0 ppm and (C) 10.0–5.0 ppm. The red and black spectral line correspond to and local samples respectively; Figure S2: A zoom on the 1H NMR extract spectra observed between; (A) 3.0–0.0 ppm, (B) 4.5–3.0 ppm, (C) 6.0–4.5 ppm and (C) 10.0–6.0 ppm. The red and black spectral line correspond to noesypr1d and zg30 samples respectively.
