# Supplementary material for: Determination of the Geographical Origin of Maltese Honey Using 1H NMR Fingerprinting
Source: Foods. 2020 Oct 13;9(10):1455. doi: 10.3390/foods9101455 (PMC7601949; doi:10.3390/foods9101455)
Supplement: Supplementary file 1 [file foods-09-01455-s001.pdf]

Table S1: A list the location and the botanical origin of each local honey sample used in this study.

Local honey			
	**Sample Code**	**Location Origin**	**Botanical Origin**
1.	AGOB	Birzebbuga	wild thyme
2.	AGT	Mellieha	wild thyme
3.	BCE	Siggiewi	carob and eucalyptus
4.	CPM	Safi	multifloral
5.	DSW	Siggiewi	wild thyme
6.	E1	Siggiewi	eucalyptus
7.	E2	Fawwara	eucalyptus
8.	G1	Fawwara	multifloral
9.	G2	Wardija	thyme
10.	G3	Wardija	multifloral
11.	G4	Mgarr	thyme
12.	G5	Mellieha	multifloral
13.	G6	Zebbiegh	multifloral
14.	G7	Gargur	multifloral
15.	G8	Gargur	thyme
16.	G9	Marsalforn	multifloral
17.	GC	Ghasri	multifloral
18.	GHB	Ghasri	multifloral
19.	GHPA	Nadur	carob
20	JPH	Nadur	Thyme
21.	MHFM	Ghajnielem	multifloral
22.	MSG	Nadur	carob and Eucalyptus
23.	MT5	Xaghra	multifloral
24.	NZFS	Kercem	multifloral
25.	NZFT	Kercem	Thyme
26.	RGEC	Mellieha	carob and Eucalyptus
27.	RGT	Mellieha	Thyme
28.	SBG	Kercem	multifloral
29.	TPM	Xewkija	multifloral
30.	WT	Zebbug	thyme
31.	ZM	Zebbug	multifloral

Table S2: A list the location and the botanical origin of each non-local honey sample used in this study.

	Non-local honey		
	**Sample Code**	**Location Origin**	**Botanical Origin**
1.	AU	Austria	combhoney
2.	DL	Germany	multifloral
3.	FR1	France	multifloral
4.	FR2	France	multifloral
5.	FRC	France	chestnut
6.	GPM1	Blended	multifloral
7.	GRC1	Greece	thyme
8.	GRC2	Greece	multifloral
9.	GRC3	Greece	multifloral
10.	GRC4	Greece	multifloral
11.	GRC5	Greece	multifloral
12.	GRC6	Greece	multifloral
13.	GRC7	Greece	thyme
14.	ITC	Italy	wildflower
15.	IWIGR	France	multifloral
16.	IW2GR	Germany	multifloral
17.	IW2IT	Italy	multifloral
18.	IW3FRI	France	multifloral
19.	IW4GR	Germany	multifloral
20	IW4JP	Japan	multifloral
21.	IW5AU	Austria	multifloral
22.	IW6	France	multifloral
23.	IW7SP	Finland	eucalyptus
24.	IW8GRC	Greece	multifloral
25.	IW9	Finland	multifloral
26.	IW10FRC	France	multifloral
27.	IW11FR	France	multifloral
28.	IW12FR	France	multifloral
29.	IW13	USA	multifloral
30.	M2	Italy	multifloral
31.	M3	Italy	orange
32.	M4	Italy	Acacia plant
33.	M5	Italy	multifloral
34.	MF	Italy	multifloral
35.	SC-CMF	Italy	multifloral
36.	SC.MZ	Italy	eucalyptus

37.	SC-MM	Italy	multifloral
38.	SC-MS	Italy	sulla flower
39.	SC-MT	Italy	thyme
40.	SC-MZ	Italy	Zagara flower
41.	SC-PA	Italy	Aneto
42.	SC-PE	Italy	Eucalyptus
43.	SC-PM	Italy	multifloral
44.	SC-PO	Italy	orange
45.	SC-PT	Italy	thyme
46.	SW37	Italy	multifloral
47.	TF	Zambia	multifloral
48.	Y1	Yorkshiire	multifloral

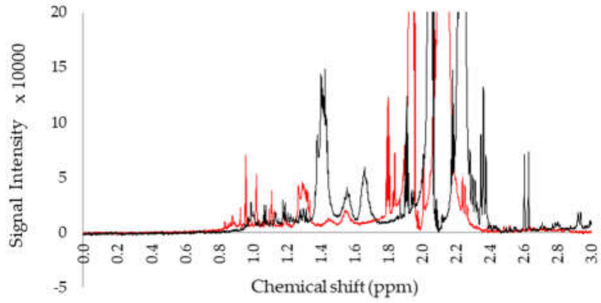

(A)

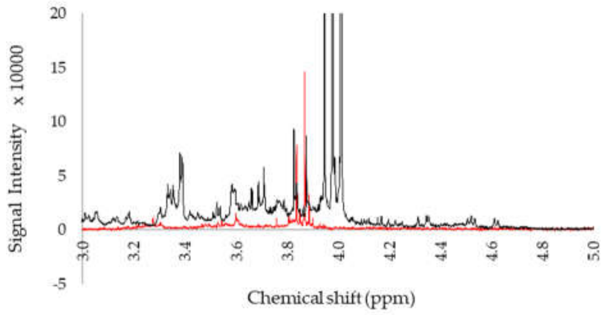

(B)

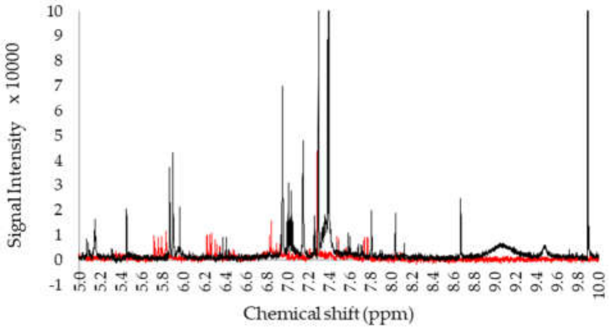

(C)

Figure S1: A zoom on the ^{1}H NMR phenolic extract spectra observed between; (A) 3.0 - 0.0 ppm, B) 5.0 – 3.0 ppm and (C) 10.0 – 5.0 ppm. The red and black spectral line correspond to non local and local samples respectively.

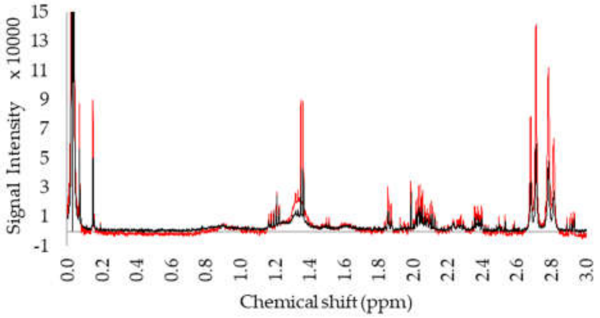

(A)

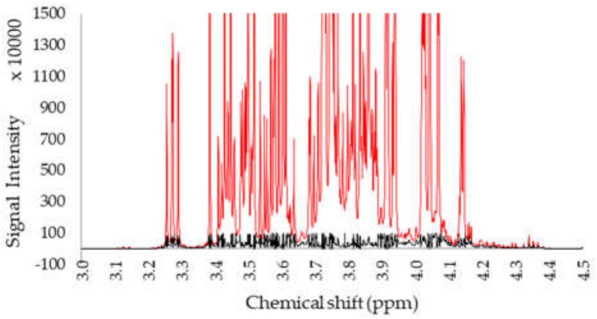

(B)

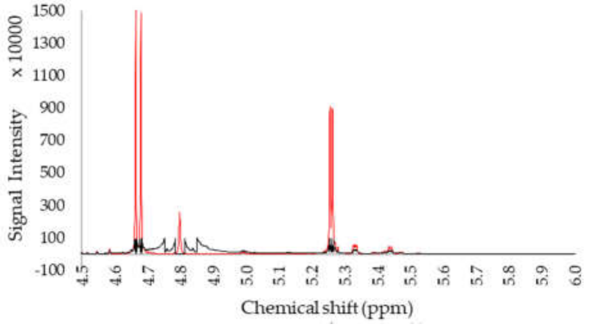

(C)

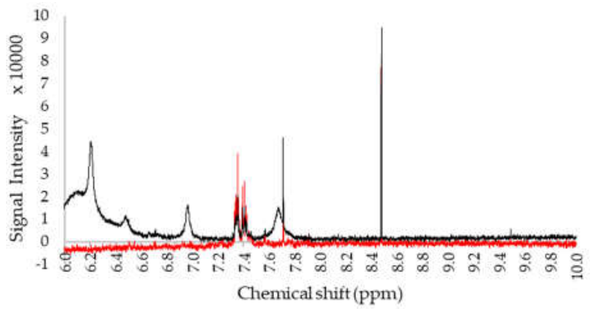

(D)

Figure S2: A zoom on the ^{1}H NMR extract spectra observed between; (A) 3.0 - 0.0 ppm, B) 4.5 – 3.0 ppm, C) 6.0 – 4.5 ppm and (C) 10.0 – 6.0 ppm. The red and black spectral line correspond to noesypr1d and zg30 samples respectively.
